# The impact of social isolation and loneliness on the well-being of carers of a person with dementia in aotearoa New Zealand

**DOI:** 10.1177/14713012241279683

**Published:** 2024-08-30

**Authors:** Kirsten Robertson, Maree Thyne, Rob Thomson, Leah Watkins

**Affiliations:** Department of Marketing, Dunedin, New Zealand; Department of Marketing, 2495 University of Otago, Dunedin, New Zealand; Department of Marketing, Dunedin, New Zealand; Department of Marketing, Dunedin, New Zealand

**Keywords:** alzheimers, loneliness, dementia, informal caregiver’s, isolation, well-being

## Abstract

Dementia is a leading cause of disability, and as the population ages, there will be a greater need for friends and family to care for people with Dementia. Unfortunately, informal care for a person with dementia is associated with poor psychological and physical health and lower quality of life of the caregiver. The aim of the present study was to understand how to best support caregivers within their communities by examining their experience of loneliness, isolation, and their relationship with well-being. The study used a representative sample of the New Zealand population in terms of ethnicity, age, gender, education, and income and asked people if they were a primary caregiver of a person with Alzheimer’s Disease or related disorder. Both loneliness and isolation were linked to overall well-being; however, loneliness was a stronger predictor of satisfaction with relationships and feeling part of one’s community. The findings highlight the importance of examining the multi-factorial constructs of social connectedness and question research attributing loneliness solely to reduced social involvement. As such, interventions for caregivers of a person with dementia need to target feelings of loneliness as well as their social isolation.

## Background

Dementia is a leading cause of disability. Over 55 million people live with dementia globally (World Health Organisation, 2023). This will only increase with the prevalence of dementia predicted to affect 153 million people by 2050 (Nichols et al., 2022). Similarly, in Aotearoa New Zealand, the number of aged individuals is estimated to double in the next two decades (Alzheimer’s New Zealand, 2020; Parr-Brownlie et al., 2020). Consequently, there will be a greater need for people to care and support persons with dementia. This care is primarily provided by adult children and partners as informal caregiving (Lapsley et al., 2020), and may allow the person with dementia to age in their own home, rather than in residential care, which is associated with improved well-being and quality of life (Fæø et al., 2019; Olsen et al., 2016; Wiles et al., 2012).

Although there are benefits of informal carers to the person with dementia, and caregiving can be rewarding through strengthening the relationship between the caregiver and the person with dementia (Lindeza et al., 2020; Quinn & Toms, 2019), this is a difficult role. Providing informal care of a person with dementia is associated with poor psychological and physical health (Collins & Kishita, 2020; del-Pino-Casado et al., 2021; Gilhooly et al., 2016; Gilsenan et al., 2023), significant physical and social burdens (Mansfield et al., 2023), and lower quality of life (Koyama et al., 2017). As such, caregivers of a person with dementia have been described as “the invisible second patients” (Brodaty & Donkin, 2009) and the WHO has declared support for carers of a person with dementia as a health priority (World Health Organisation, 2023). This study explores the wellbeing and social connectedness of caregivers of people with dementia.

A lack of social relationships is linked to all-cause mortality (Granbo et al., 2019; Naito et al., 2023), and is especially harmful to older adults (Gerst-Emerson & Jayawardhana, 2015; Lennartsson et al., 2022). For caregivers of a person with dementia, social relationships and social activities may be hindered (Beeson, 2003; Clark & Bond, 2000; Vasileiou et al., 2017), due to being housebound (Granbo et al., 2019), socially excluded (Greenwood et al., 2018), a desire for privacy, and not wanting to be a burden (Robertson et al., 2022). Existing social services have tried to reduce loneliness amongst caregivers by providing support groups, however, a systematic review found support groups were generally ineffective and did not increase perceptions of social connectedness (Cheng & Zhang, 2020). Consequently, there is a need for research to determine how to address loneliness and effectively facilitate social connection amongst caregivers (Lee et al., 2022; Van Orden & Heffner, 2022; Velloze et al., 2022).

Social connectedness is often a catch-all term for a variety of multi-factorial constructs that are frequently used interchangeably or not defined (Courtin & Knapp, 2017; Holt-Lunstad et al., 2017). A recent review found that although studies were consistent in their definitions of loneliness, how researcher’s defined social isolation differed across studies (Guan et al., 2023). Researchers describe social isolation as a lack of social interactions (Holt-Lunstad et al., 2017), whereas loneliness is characterised by the perception of being socially disconnected (Perlman & Peplau, 1981) or by not having the quality of social relationships one desires (Heinrich & Gullone, 2006). Other researchers describe social isolation as a construct comprising of both objective social disconnection and subjective feelings of loneliness (Cornwell & Waite, 2009; Newall & Menec, 2019). Although these concepts are related and are both associated with psychological distress (Courtin & Knapp, 2017), they are only moderately associated (Dahlberg et al., 2018; Taylor, 2020) and should be considered separately as people can feel lonely even though they have plenty of social interactions and vice versa (Newall & Menec, 2019; Victor et al., 2021). Research also suggests that loneliness and social isolation may have different effects on health as loneliness is a stronger predictor of psychological outcomes whereas social isolation is more predictive of mortality risk (Hong et al., 2023).

Qualitative research with caregivers of people with dementia reveals they identify different components of social disconnection (Lindeza et al., 2020; Vasileiou et al., 2017), such as decreased social interaction, feelings of helplessness and a sense of sole responsibility. Research has underscored the prevalence of a lack of connectedness among caregivers of persons with dementia. A cohort study conducted in the UK discovered that 44% of caregivers experienced a moderate level of disconnection (social loneliness/isolation and emotional loneliness), with 18% reporting severe disconnection (Victor et al., 2021). Other longitudinal studies have corroborated these findings, revealing that connectedness, encompassing feelings of lacking companionship, feeling left out, and isolation from others, is related to depression (Saadi et al., 2021). Despite these insights into the social disconnection experienced by caregivers, there remains a dearth of knowledge regarding their experiences of isolation and loneliness, and how these affect their wellbeing.

This quantitative study examined whether caregivers of a person with dementia were more likely than non-caregivers to experience social isolation and felt loneliness in a population based New Zealand study. Based on previous research it was expected that a lack of connection would be associated with reduced well-being (Victor et al., 2021) but whether the relationship would differ for loneliness (emotional state) versus isolation (more circumstantial) was unknown due to the two constructs often being measured together. Research amongst the wider population shows the quality of social relationships varies with gender, age, income, and deprivation (Geerling & Diener, 2020; Hawthorne, 2008; Lay-Yee et al., 2022; Lee et al., 2022), and income is the strongest predictor of well-being (Geerling & Diener, 2020). Consequently, these demographics were controlled for in this current study.

## Method

### Participants

The data used in this study was gathered from the 2020 New Zealand Lifestyles Survey, which was a representative sample of 1643 individuals (48% female), 18 years and older. Quota sampling was employed to achieve a sample representative of the Aotearoa New Zealand population in terms of ethnicity, age, gender, education, and income. The demographics of the sample are presented in Table 1. The current research focussed on the experience of caregivers in the general population and acknowledge that the methodologies and measures used may not be appropriate for Māori and other ethnic groups. This will be addressed in future research.

**Table 1.** Comparison of demographic information in caregivers and non-caregivers of a person with dementia.

Variable	Caregivers	Non-caregivers	Total
Age**			
*Mean (SD) age in years*	42.8 (18.4)	47.2 (18.6)	46.8 (18.6)
Gender			
*Male*	62 (48.8%)	725 (48.3%)	787 (48.3%)
*Female*	65 (51.2%)	777 (51.7%)	842 (51.7%)
Ethnicity			
*NZ European*	76 (58.5%)	1012 (66.9%)	1088 (66.2%)
*Māori*	17 (13.1%)	161 (10.6%)	178 (10.8%)
*Other*	37 (28.4%)	340 (22.5%)	377 (23.0%)
Education			
*With university qualification*	44 (33.8%)	558 (36.9%)	602 (36.6%)
*Without university qualification*	86 (66.2%)	955 (63.1%)	1041 (63.4%)
Annual income			
*Less than or equal 50,000 NZD*	73 (56.2%)	771 (51.0%)	844 (51.4%)
*More than 50,000 NZD*	57 (43.8%)	742 (49.0%)	799 (48.6%)
Living environment***			
*Urban*	86 (66.2%)	1175 (77.7%)	1261 (76.7%)
*Rural*	32 (24.6%)	179 (11.8%)	211 (12.8%)
*Semi-rural*	12 (9.2%)	159 (10.5%)	171 (10.4%)
Employment			
*Employed*	79 (60.8%)	821 (54.3%)	900 (54.8%)
*Studying*	7 (5.4%)	91 (6.0%)	98 (6.0%)
*Not employed*	44 (33.8%)	601 (39.7%)	645 (39.3%)
Housing			
*Own with mortgage*	37 (28.5%)	409 (27.0%)	446 (27.1%)
*Own mortgage free*	41 (31.5%)	450 (29.8%)	491 (29.9%)
*Rent*	52 (40.0%)	654 (43.2%)	706 (43.0%)
Children under 18 living at home			
*Yes*	40 (30.8%)	438 (28.9%)	478 (29.1%)
*No*	90 (69.2%)	1075 (71.1%)	1165 (70.9%)

Income was classified as above or below the national median ($50,000NZD). Overall, there was an equal gender split, the sample were predominantly of European ethnicity (66%) and ranged from 18-90 years (*M* = 46.8 years, *SD* = 18.6). The majority (77%) resided in urban areas, 37% had a university degree, and 55% were in employment. Of the sample, 130 respondents indicated that they were a primary caregiver of a person with dementia. Although the mean age for caregivers was lower than non-caregivers, and there were differences in living environment, there was no relationship between these variables and the outcome measures.

### Measures

#### Well-being

Well-being was measured using two scales, The Flourishing Scale, and The Personal Well-being Index. The eight-item Flourishing Scale offers a single score for psychological well-being (Diener et al., 2010). Responses were on a seven-point likert scale from 1 (strongly disagree) to 7 (strongly agree). The scale has been found to be a reliable and valid measure of psychological functioning (Pruchno et al., 2008) correlating well with other measures of psychological well-being (Hone et al., 2014). The reliability for the eight items used in the present study was good (Cronbach Alpha = 0.91). To determine levels of flourishing the total scores were recoded as an ordinal variable with three categories: low (8–38), medium (39–46), and high (47–56), and approximately one-third of the participants were captured in each level.

The seven-item Personal Well-being Index scale (Cummins et al., 2003) measures satisfaction across seven life domains, including standard of living, health, accomplishing in life, relationships, safety, sense of community, and future security. Theoretically, the first level deconstruction of the global question, “How satisfied are you with your life as a whole?” is represented by these seven areas. Items can either be used individually as separate variables or summed to obtain an overall average of subjective wellbeing. Responses are made on a 11-point satisfaction scale from 1 (no satisfaction at all) to 11 (completely satisfied). The scale is commonly used to measure subjective well-being (International Well-being International Wellbeing Group, 2013). Two domains pertinent to this study were used: relationship satisfaction (e.g., “How satisfied are you with your personal relationships?”) and Sense of community (e.g., “How satisfied are you with feeling part of your community?”). The scores were recoded as an ordinal variable with three categories: low (0–5), medium (6–7), and high (8–10), and approximately one-third of the participants were captured in each level.

#### Loneliness and isolation

Isolation and loneliness were assessed using the six-item Friendship Scale (Hawthorne, 2006), which has been used to examine social connectedness among caregivers in Australia (Poon et al., 2022). Traditionally, the scale is treated as a single dimension, gauging perceived social isolation (e.g., “I found it easy to connect with others when I needed to”) and feelings of loneliness (e.g., “When with other people, I felt separate from them”; Hawthorne, 2008), however, the friendship scale has been found to have two dimensions. Three items measure perceived social isolation, e.g., “*I found it easy to get in touch with others when I needed to*” and three items measure felt loneliness, e.g., “*When with other people I felt separate from them”* (Hawthorne, 2008). These two dimensions were employed in the current study. Participants were asked to reflect on their experiences over the past four weeks and rate the degree to which each statement applied to them on a five-point Likert scale from 1 (almost always) to 5 (not at all). Higher responses corresponded to greater levels of loneliness and increased isolation. The total scores were recoded into an ordinal variable with three categories: low (3–4), medium (5–7), and high (8–15), and roughly one-third of the participants fell into each of these categories.

To investigate using the scale as two distinct sub-scales (isolation and loneliness) a Principal Component Factor Analysis with Promax rotation to unveil the underlying factor structure and identify key dimensions was conducted. This analysis, which included the six Friendship scale items, accounted for 73% of the variance. The rotated factor loadings following the Promax rotation are presented in Table 2 with two factors identified as Loneliness and Social Isolation. A reliability analysis using Cronbach’s alpha indicated robust alpha values for both Factor 1 (α = 0.87) and Factor 2 (α = 0.76), indicating strong internal consistency. Notably, the items closely align with those employed in prior studies to assess isolation (e.g., sensations of being excluded) and loneliness (e.g., the presence of a special person during times of need, Matthews et al., 2016). The correlation between isolation and loneliness demonstrated a moderate association (r = 0.43).

**Table 2.** Rotated loadings for Factor 1 (Loneliness) and Factor 2 (Social isolation) of the Friendship Scale.

	Factor 1: Loneliness	Factor 2: Social isolation
It has been easy to relate to others	.369	**.816**
I had someone to share my feelings with	.298	**.803**
I found it easy to get in touch with others when I needed to	.387	**.850**
I felt isolated from other people	**.909**	.410
When with other people I felt separate from them	**.861**	.300
I felt alone & friendless	**.889**	.431

## Results

The mean scores for caregivers and non-caregivers on the Flourishing Scale, the subscales of the Personal Wellbeing Index (Relationship satisfaction & Feeling part of the community) and the Friendship Scale (Isolation & Loneliness) were calculated and are presented in Table 3. It was found that caregivers scored lower on the Flourishing Scale (*X* = 40.3, *SE* = 0.7) than non-caregivers (*X* = 41.8, *SE* = 0.2) (*F*(1,1641) = 4.16, *p* < .05), however there was not a significant difference for the Personal Wellbeing Index nor the subscales. Although caregivers did not feel more isolated, they did feel more loneliness (*X* = 7.8, *SE* = 0.3) than non-caregivers (*X* = 7.8, *SE* = 0.3) (*F*(1,1641) = 19.64, *p* < .001).

**Table 3.** Mean scores (S.E.) on measures of wellbeing, isolation and loneliness for caregivers and non-caregivers.

	Caregiver	Non-caregiver
Flourishing Scale*	40.3 (0.7)	41.8 (0.2)
Personal wellbeing index		
Relationships satisfaction	7.7 (0.2)	7.9 (0.1)
Part of community	7.0 (0.1)	7.2 (0.2)
Friendship Scale		
Isolation	7.7 (0.2)	7.5 (0.1)
Loneliness***	7.8 (0.3)	6.4 (0.1)

To examine the association between loneliness, caregiver status, and potential covariates (income, age, gender, relationship status, and education), an ordinal regression for loneliness was calculated. The outcome variable, loneliness, was categorized into three ordinal levels. The ordinal regression model demonstrated robust goodness of fit, underscored by the −2 Log-Likelihood statistic χ^2^ (9) = 306.48, *p* < .001), and the Nagelkerke R-squared value (*R*^2^ = 0.193), signifying its ability to explain a substantial portion of the observed variance. The parameter estimates of Caregivers and covariates on Loneliness and Isolation factors of the Friendship Scale are presented in Table 4. After adjusting for income, age, gender, relationship status, and education, the odds of a Caregiver being lonely was 1.77 (95% CI, 1.24–2.53) times more likely than a non-caregiver (Wald χ^2^ (1) = 9.91, *p* < .01).

**Table 4.** Parameter estimates of Caregivers and covariates on Loneliness and Isolation factors of the Friendship Scale.

	B	SE	Wald	df	Sig	Exp_B	95% CI lower	95% CI upper
Loneliness								
Caregiver	.57	.18	9.90	1	.002	1.77	1.24	2.53
Income	−.04	.01	12.95	1	.000	.96	.94	.99
Age	−.04	.00	197.62	1	.000	.96	.95	.96
Single	.17	.12	11.47	1	.149	1.19	.94	1.50
Widowed/divorced Married (ref)	.54	.16	11.47	1	.000	1.71	1.25	2.34
Gender (female)	−.12	.10	1.41	1	.235	.89	.74	1.08
No formal qual	.24	.19	1.58	1	.209	1.27	.88	1.83
School level qual	−.10	.13	.639	1	.424	.90	.70	1.16
Tech/prof qual Uni qual (ref)	−.01	.12	.004	1	.950	.99	.79	1.26
Isolation								
Caregiver	.18	.18	.98	1	.321	1.20	0.84	1.71
Income	−.04	.01	11.62	1	.000	.96	.94	.98
Age	−.02	.00	46.37	1	.000	1.00	.97	.99
Single	.61	.12	25.12	1	.000	1.84	1.45	2.33
Widowed/divorced Married (ref)	.70	.16	18.83	1	.000	2.00	1.46	2.74
Gender (female)	−.12	.10	1.45	1	.228	.89	.73	1.08
No formal qual	.40	.19	4.36	1	.37	1.49	1.03	2.18
School level qual	.02	.13	.03	1	.874	1.02	.79	1.32
Tech/prof qual Uni qual (ref)	.18	.12	2.27	1	.132	1.20	.95	1.51

The initial analyses did not yield a significant association between caregiver status and isolation. A subsequent ordinal regression exploring whether the relationship between caregiver status and isolation differed when demographics were controlled for revealed caregiver status to remain non-significant Wald χ^2^ (1) = .983, *p* = .321. However, the ordinal regression model exhibited a robust fit to the data by the −2 Log-Likelihood statistic χ^2^ (9) = 150.46, *p* < .001 and the Nagelkerke R-squared value (*R*^2^ = .101).

To examine the association between loneliness (three ordinal levels), and isolation (three ordinal levels), on flourishing (three ordinal levels), while controlling for income (continuous) and caregiver status, an ordinal regression was calculated. The ordinal regression model demonstrated robust goodness of fit, underscored by the −2 Log-Likelihood statistic χ^2^ (6) = 419, *p* < .001), and the Nagelkerke R-squared value (*R*^2^ = .254), signifying its ability to explain a substantial portion of the observed variance. The parameter estimates of Loneliness and Isolation on Flourishing, Personal relationships and Feeling part of the community factors of the Personal Wellbeing Index are presented in Table 5. After adjusting for income and caregiver status, Flourishing was found to be significantly more likely in individuals who were less lonely, and amongst those who were less isolated. Isolation was the strongest predictor of flourishing (Wald = 87.04, *p* < .001). Individuals who experienced low levels of Isolation were 3.4 times more likely to report higher flourishing than those who experienced high levels of Isolation (95% CI: 2.6–4.4).

**Table 5.** Parameter estimates of loneliness and isolation on flourishing, personal relationships and feeling part of the community factors of the personal wellbeing index.

	B	SE	Wald	df	Sig	Exp_B	95% CI lower	95% CI upper
Flourishing								
Loneliness (low)	1.22	.13	87.04	1	.000	3.40	2.63	4.40
Loneliness (med)	.702	.12	35.03	1	.000	2.02	1.60	2.54
Loneliness (high, ref)								
Isolation (low)	1.68	.15	121.08	1	.000	5.35	3.97	7.21
Isolation (med)	.82	.11	55.02	1	.000	2.26	1.82	2.81
Isolation (high, ref)								
Income	.04	.01	13.43	1	.000	1.04	1.02	1.06
Caregiver	.17	.18	.916	1	.338	.84	.59	1.20
Personal relationships								
Loneliness (low)	1.66	.14	141.23	1	.000	5.27	4.01	6.94
Loneliness (med)	.823	.12	47.35	1	.000	2.28	1.80	2.88
Loneliness (high, ref)								
Isolation (low)	1.62	.17	88.92	1	.000	5.07	3.619	7.11
Isolation (med)	.738	.11	42.77	1	.000	2.09	1.677	2.61
Isolation (high, ref)								
Income	.04	.01	13.43	1	.000	1.04	1.018	1.06
Caregiver	.17	.18	.916	1	.338	.84	.590	1.20
Feeling part of community								
Loneliness (low)	1.39	.13	106.58	1	.000	4.00	3.07	5.20
Loneliness (med)	.87	.12	54.92	1	.000	2.395	1.90	3.02
Loneliness (high, ref)								
Isolation (low)	.78	.15	26.07	1	.000	2.186	1.62	2.95
Isolation (med)	.52	.11	22.29	1	.000	1.688	1.36	2.10
Isolation (high, ref)								
Income	.04	.01	16.74	1	.000	1.041	1.02	1.06
Caregiver	.32	.18	3.14	1	.077	.729	.514	1.03

Ordinal regression was used to examine the association between loneliness (three ordinal levels), and Isolation (three ordinal levels), to the dependent variable satisfaction with personal relationships (three ordinal levels), while controlling for income (continuous) and caregiver status. The ordinal regression model demonstrated robust goodness of fit, underscored by the −2 Log-Likelihood statistic χ^2^ (6) = 448.562, *p* < .001), and the Nagelkerke R-squared value (*R*^2^ = .274). After adjusting for income and caregiver status, satisfaction with personal relationships was found to be significantly more likely in individuals who were less lonely, and amongst those who were less isolated. The ordinal regression analysis revealed that loneliness was the strongest predictor of satisfaction with relationships (Wald = 141.23, *p* < .001). Individuals who experienced low levels of loneliness were 5.3 times more likely to report higher satisfaction with their relationships than those who experienced high levels of loneliness (95% CI: 4.0–7.0).

Finally, ordinal regression was used to examine the association between loneliness (three ordinal levels), and Isolation (three ordinal levels), to the dependent variable satisfaction with feeling part of the community (three ordinal levels), while controlling for income (continuous) and caregiver status. The ordinal regression model demonstrated robust goodness of fit, underscored by the −2 Log-Likelihood statistic χ^2^ (6) = 275.742, *p* < .001), and the Nagelkerke R-squared value (*R*^2^ = .176). After adjusting for income and caregiver status, satisfaction with feeling part of the community was significantly more likely in individuals who were less lonely, and amongst those who were less isolated, however, the relationship was strongest for those who were less lonely (Wald = 106.58, *p* < .001). Individuals who experienced low levels of loneliness were 4.0 times more likely to report higher satisfaction with their community than those who experienced high levels of loneliness (95% CI: 3.07–5.2).

## Discussion

The present study examined whether caregivers of a person with dementia were more likely than non-caregivers to experience social isolation and feel loneliness, and how these affect their well-being. Although there was no significant difference in participant’s experience of social isolation, caregivers were significantly more likely to feel lonely (e.g., alone, and friendless). Caregivers also scored lower on the Flourishing Scale than non-caregivers. The examination of well-being revealed both loneliness and isolation contribute to reduced happiness. However, when satisfaction with relationships and feeling part of the community were examined, loneliness was found to be a stronger predictor of satisfaction than isolation.

Unlike other studies that found that caregivers were more socially disconnected than non-caregivers (Saadi et al., 2021; Victor et al., 2021), there were no differences in this cohort. The experience of loneliness by caregivers in the present study echoes the feelings of loneliness and helplessness described in qualitative studies (Lindeza et al., 2020; Vasileiou et al., 2017). This suggests that loneliness and isolation are distinct constructs (Newall & Menec, 2019; Victor et al., 2021), and questions research attributing loneliness solely to reduced social involvement (Clark & Bond, 2000). These findings extend previous research (Saadi et al., 2021; Victor et al., 2021) by teasing apart loneliness and isolation, and suggest a need for interventions to focus on loneliness more so than social isolation, addressing the call for greater understanding of the determinants of connectedness to guide interventions (Courtin & Knapp, 2017; Jeste et al., 2021; Van Orden & Heffner, 2022).

Interventions such as support groups, which tend to fall short of addressing social connectedness (Velloze et al., 2022) might partially address social isolation through fostering social interaction but may not alleviate the emotional experience of loneliness. Encouraging caregivers to engage in social activities to improve their well-being may only be effective if these focus on establishing emotional connections to overcome loneliness. This suggests that further interventions are needed to establish emotional connection to overcome loneliness.

We believe our findings reflect a need for greater understanding of dementia and empathy towards people living with dementia amongst the wider community to reduce caregivers’ feelings of being alone. Unfortunately, our communities are not dementia friendly; stigma towards people with dementia and their family, leads to distancing and subtle condemnation (Vasileiou et al., 2017). The resulting detrimental effects include decreased quality of life, poor physical and mental health, caregiver burden and social isolation, amongst other negative impacts (Burgener et al., 2015). Western biomedical models have dominated understandings of dementia and have largely focussed on just the person with dementia (or maybe their care partner); however, dementia affects the whole whānau (family) (Dudley et al., 2019). Echoing Holt-Lunstad et al.’s. (2017) recommendation, we argue loneliness should be addressed at the societal level. For example, the UK’s campaign to end loneliness in older adults has been directed at policymakers, organisations, and individuals to increase understanding of loneliness and promote action to reduce it (Ferguson, 2011). The top-down campaign, which involved a coalition of individuals and organisations, has had a significant impact on media interpretation, practice, and policy around loneliness (Goodman & Symons, 2013). Following the campaign, several organisations begun targeting loneliness including the “Be More Us Campaign”, which inspires and empowers individuals to connect with one another. We suggest such campaigns should also focus on vulnerable sub-populations, specifically carers of a person with dementia, rather than the general population.

There are several limitations within this study. As with previous studies, a self-report item was used to measure caregiving, and the level of the care being provided was not measured (Saadi et al., 2021). Moreover, it is possible that the experience of loneliness differs throughout the caregiving journey, and we were not able to capture this with our classification. Future research needs to better understand the experience of loneliness and isolation in caregivers as the person with dementia’s condition changes and investigate specific strategies to improve well-being amongst carers.

Another limitation of the research is that the measures used in the present study are designed from a western perspective and reflect western values and beliefs. Although approximately 11% of the respondents were Māori, this Indigenous group was underrepresented in the study, and the measures used may not be appropriate for this group. Current research is taking a kaupapa Māori (Indigenous led) approach in building partnerships with local Indigenous groups to identify the issues, challenges, and strengths specific to Māori whānau with dementia (mate wareware). Future research also needs to examine experiences of dementia in different cultures (Taylor et al., 2023), and how dementia impacts the whole family. Some cultures are more likely to emphasise informal caregiving within extended family structures instead of using residential care (Lapsley et al., 2020). Consequently, a comprehensive and culturally sensitive approach is essential to address these complex issues. Gaining a better understanding of whānau experiences will ensure research integrity, meets the needs of local communities, and will help inform support to be whānau-led rather than service-led or government-led.

As the number of people with dementia continues to increase, understanding how to support the caregivers will be increasingly important. Through greater understanding of the psychosocial needs of caregivers and family of individuals with dementia, we can develop tailored support programmes to address social needs, enhance their well-being and help them better support the person with dementia and their communities.

## Data Availability

The data that support the findings of this study are available on request from the corresponding author. The data are not publicly available due to privacy and ethical restrictions.*
